# Correction: Barkjohn et al. Correction and Accuracy of PurpleAir PM_2.5_ Measurements for Extreme Wildfire Smoke. *Sensors* 2022, *22*, 9669

**DOI:** 10.3390/s24247871

**Published:** 2024-12-10

**Authors:** Karoline K. Barkjohn, Amara L. Holder, Samuel G. Frederick, Andrea L. Clements

**Affiliations:** 1 US Environmental Protection Agency Office of Research and Development, Research Triangle Park, Durham, NC 27711, USA; 2 Former ORAU Student Services Contractor, US Environmental Protection Agency Office of Research and Development, Research Triangle Park, Durham, NC 27711, USA; 3 Currently Department of Atmospheric Sciences, University of Illinois Urbana-Champaign, Urbana, IL 61801, USA

## Figure Correction

In the original publication [1], Figure 4 has been updated to show both Nilson cf_atm and cf_1, as indicated below.

**Figure 4:**
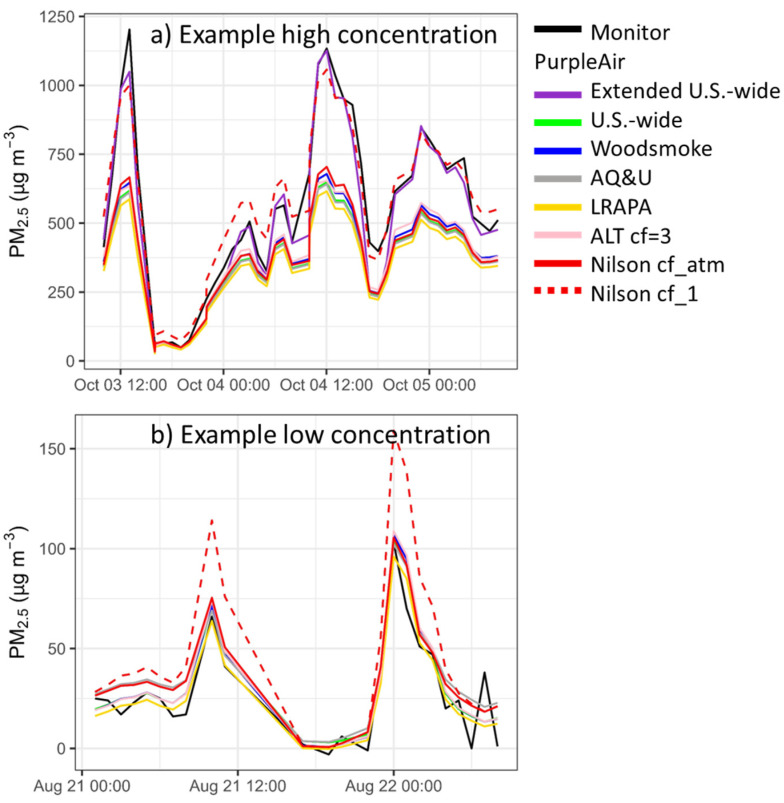
Example time series from the Forks of Salmon, CA, showing the data that were corrected based on different corrections on the PurpleAir map (Woodsmoke, AQandU, LRAPA, ALT cf = 3) and in the literature (Nilson cf_atm and cf_1).

Figure 5 has been updated to correct an error in calculating the high-concentration extended line and changing Nilson to cf_atm, as indicated below.

**Figure 5:**
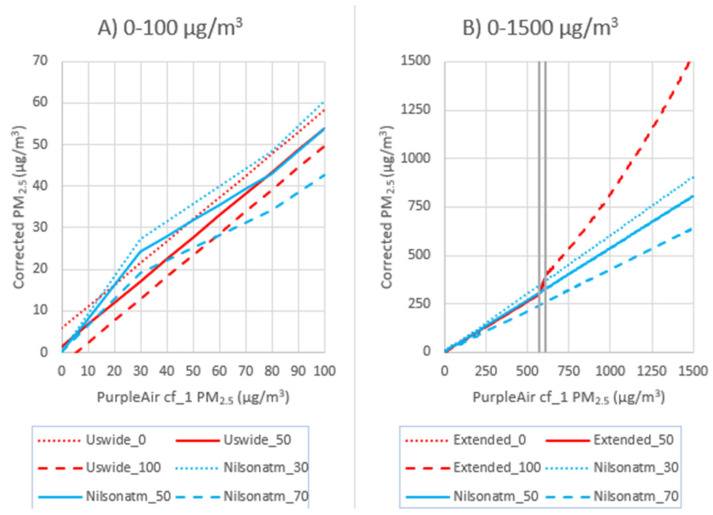
Disagreement between Nilson cf_atm (actually used in Nilson’s paper based on their corrigendum) and the extended US-wide correction, plotted over the range of RH used in each correction, shown at low concentrations (**A**) and over the full range (**B**) with gray vertical lines showing the transition zone from the US-wide correction to the quadradic fit.

## Table Correction

Table 3 has been updated as shown below.

**Table 3:** Correction equations that were considered.

Model	Fit	Equation
A	U.S. correction	PM_2.5_ = 0.524 × PA_cf_1_ − 0.0862 × RH + 5.75
B	Quadratic	PM_2.5_ = a × PA_cf_1_^2^ + b × PA_cf_1_ + c
C	Cubic	PM_2.5_ = a × PA_cf_1_^3^ + b × PA_cf_1_^2^ + c × PA_cf_1_ + d
D	Quadratic + RH	PM_2.5_ = a × PA_cf_1_^2^ + b × PA_cf_1_ + c + d × RH
E	Quadratic PM × RH	PM_2.5_ = a × PA_cf_1_^2^ + b × PA_cf_1_ +c + d × RH + e × PA_cf_1_ × RH
F	RH growth, Nilson [28] *	${\mathrm{PA}}_{\mathrm{cf}\_1}/\mathrm{monitor}=a+\frac{b}{\left(\frac{100}{RH}-1\right)}$ (RH limited 30–70%)

* After the initial publication of this paper, a corrigendum was released for Nilson et al.’s 2022 correction work [28], revealing that they had actually used cf_atm data. Cf_1 data have been used for Nilson data throughout the paper, except in some figures, where it is explicitly noted as Nilson atm.

## Text Correction

1. The second paragraph in Section 3.5 has been updated to the following:

For this paper, we also considered the Nilson equation [28]. This equation is used in a PM_2.5_ map of Canada, showing the FEM monitors, along with the PurpleAir and AQ Egg sensors (https://cyclone.unbc.ca/aqmap/, accessed on 26 July 2022). After the initial publication of this paper, a corrigendum was released for Nilson et al.’s 2022 correction work [28], revealing that they had incorrectly reported the label of the PM_2.5_ data they had used. Nilson et al. reported that they had used cf_1 data when, in fact, they had used cf_atm data. The Nilson correction as intended with cf_atm data is in line with other corrections used for low-concentration PurpleAir data (Figure 4). The Nilson correction using cf_atm performs similarly to our US-wide correction [17] at low concentrations (Figure 5A), but at high concentrations shows strong underestimations compared to the extended US-wide correction (Figure 5B). Therefore, the Nilson et al. correction is not appropriate for extreme smoke. Note that Figure 5 shows both equations plotted against the cf_1 data where the cf_1 and cf_atm data have a 1:1 relationship until cf_1 = 30 µg/m^3^ and then transition to a relationship of cf_atm/cf_1 = 0.66 at cf_1 = 80 µg/m^3^.

2. The third paragraph in Section 3.5 has been updated to the following:

We fitted and tested a model of a similar form (Model F, Table 3) on the cf_1 dataset in this study, which resulted in significant overestimates between the breakpoint to unhealthy values for sensitive groups (UHSG) to the breakpoint to “Hazardous” AQI (28–300 µg/m^3^, NMBE = 42% to 63%, Table S9). The equation developed on the dataset in this paper has slightly higher coefficients than the equation developed by Nilson, but the coefficients are within 10% (Figure S7 in the Supplementary Materials). The slightly higher coefficients suggest a higher PurpleAir/monitor ratio in our dataset, which is likely due to our use of cf_1 data instead of the cf_atm data used in Nilson’s paper. In addition, the RH term is slightly stronger, suggesting slightly more hygroscopic growth in our dataset. The Nilson correction does not agree well with the correction that we have developed in this paper.

3. In the second paragraph of Section 4, the word “develope” has been updated to “develop”.

## Reference Correction

Ref. [28] has been updated:

28. Nilson, B.; Jackson, P.L.; Schiller, C.L.; Parsons, M.T. Development and Evaluation of Correction Models for a Low-Cost Fine Particulate Matter Monitor. *Atmos. Meas. Tech.* **2022**, *15*, 3315–3328, Corrigendum at https://doi.org/10.5194/amt-15-3315-2022-corrigendum.

The authors apologize for any inconvenience caused and state that the scientific conclusions are unaffected. This correction was approved by the Academic Editor. The original article has been updated.
