# Supplementary material for: Correction and Accuracy of PurpleAir PM2.5 Measurements for Extreme Wildfire Smoke
Source: Sensors (Basel). 2022 Dec 10;22(24):9669. doi: 10.3390/s22249669 (PMC9784900; doi:10.3390/s22249669)
Supplement: Supplementary file 1 [file sensors-22-09669-s001.zip › sensors-2052600-supplementary.pdf]

Supplemental Information

Correction and Accuracy of PurpleAir PM2.5 Measurements for Extreme Wildfire Smoke

Karoline K. Barkjohn [1,*], Amara L. Holder [1], Samuel G. Frederick [2,3] and Andrea L. Clements [1]

[1] US Environmental Protection Agency Office of Research and Development, Research Triangle Park, Durham, NC 27711, USA
[2] Former ORAU Student Services Contractor, US Environmental Protection Agency Office of Research and Development, Research Triangle Park, Durham NC 27711, USA
[3] Currently Department of Atmospheric Sciences, University of Illinois Urbana-Champaign, Urbana, IL 61801, USA
* Correspondence: barkjohn.karoline@epa.gov

1 Removing clear outliers from the reference monitors

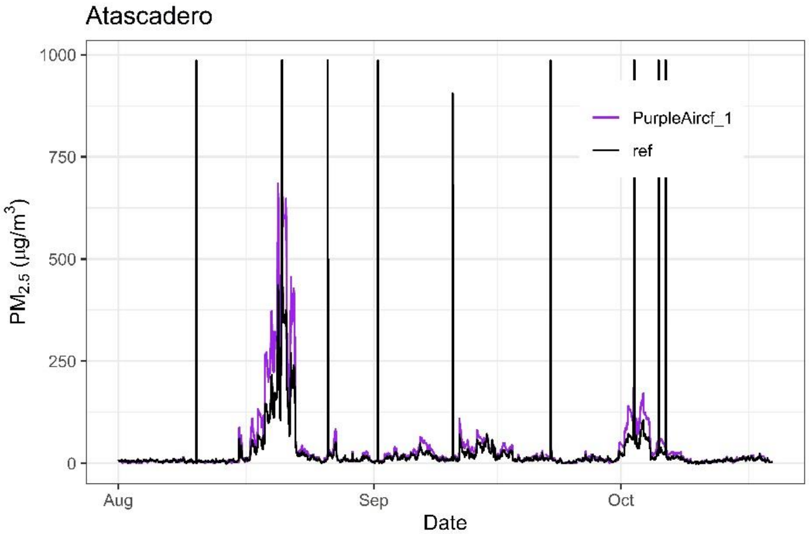

a)

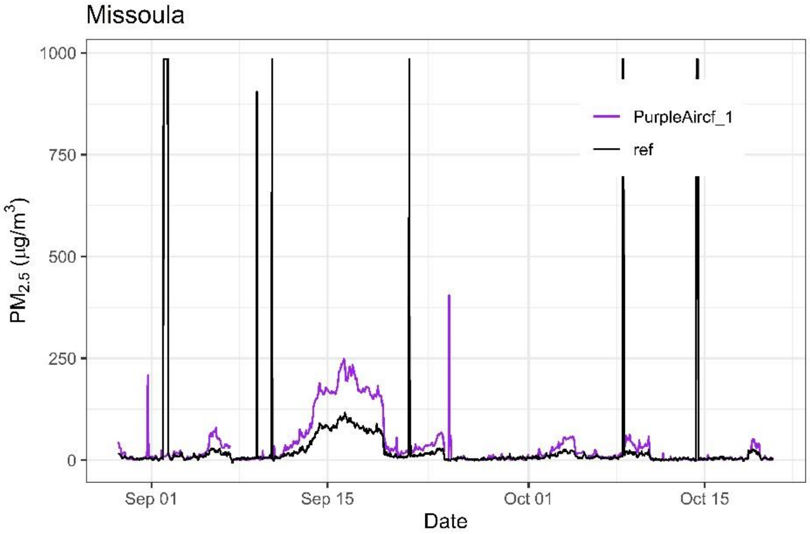

b)

Figure S 1. Clear outliers were seen in the Atascadero (a) and Missoula (b) datasets and reference points above 750 µg/m³ were removed. Many of these are instrument error codes.

2 Additional details on sensor monitor pairs evaluated

Table S 1**. Reference monitor IDs, PurpleAir names, and locations for true collocations.**

Type	City	Source	ID	PA name	Latitude	Longitude
Smoke	Happy Camp, CA	Offline	Research instrument	Offline	41.8	-123.4
	Missoula, MT	AQS	30-063-0024	Offline	46.84218	-114.021
	Oakley, UT	Offline	Research instrument	Offline	40.7	-111.3
	Pinehurst, CA	Offline	Research instrument	Offline	36.7	-119.0
	RTP, NC	AirNow Tech	37-063-0099	Offline	35.889391	-78.8742
Smoke and Ambient	Atascadero, CA	AQS	6-79-8002	Atascadero	35.49453	-120.666
Ambient	Appleton, WI	AQS	55-087-0009	Appleton	44.30738	-88.3951
	Cedar Rapids, IA	AQS	19-113-0040	Private (Linn2)	41.97677	-91.6877
	Decatur, GA	AQS	13-089-0002	Private (LTPP_GA)	33.6878	-84.2905
	Denver, CO	AQS	8-031-0026	Private (LTPP_CO)	39.77949	-105.005
	Edmond, OK	AQS	40-109-1037	Private (LTPP_OK)	35.61413	-97.4751
	Marysville, WA	AQS	53-061-1007	Marysville 7th	48.05432	-122.172
	Missoula, MT	AQS	30-063-0024	EPA 92B4	46.84218	-114.021
	Phoenix, AZ	AQS	04-013-0019	Private (LTPP_AZ)	33.48385	-112.143
	RTP, NC*	AirNow Tech	37-063-0099	Private (LTPP_NC01)	35.889391	-78.8742
	Sarasota, FL	AQS	12-115-0013	SCG Air & Water Quality_EPA Air Sensor	27.29056	-82.5072
	Topeka, KS	AQS	20-177-0013	KNI_E2	39.02427	-95.7113
	Wilmington, DE	AQS	10-003-2004	Private (LTPP_DE)	39.73944	-75.5581

Table S 2. Reference monitor types, IDs, and locations for nearby sensor monitor pairs identified on the Fire and Smoke map.

	Monitor					PurpleAir			
City	Source	ID	Monitor type	Latitude	Longitude	Name	Latitude	Longitude	distance (m)
Bend, OR	AirNow	41-017-0120	Radiance Research M903 With Heated Inlet - Nephelometry	44.06392	-121.313	BPS	44.06394	-121.313	2
Boise, ID	AirNow	16-001-0010	Met-One BAM-1020 W/PM2.5 SCC - Beta Attenuation	43.6007	-116.348	K Bar T	43.58592	-116.317	2994
El Portal	Airsis	lon_.119.784_lat_37.675_apcd.1011	E-BAM	37.675	-119.784	CARB_SMOKE_MCAPCD_Old_Elportal	37.67584	-119.77941	416
Forks of Salmon, CA	Airsis	lon_.123.331_lat_41.260_arb2.1035 ARB 1035 (Salmon)	E-BAM	41.260	123.331	Forks of Salmon	37.64591	-118.967	7
Hoopa, CA	Airsis	lon_.123.675_lat_41.047_arb2.1025 ARB 1025 (Hoopa 2)	E-BAM	41.047	123.675	Hoopa HS	41.047067	-123.675	7
Keeler, CA	AirNow	6-027-1003	Thermo Scientific TEOM 1400 FDMS or 1405 8500C FDMS w/VSCC - FDMS Gravimetric	36.48782	-117.871	CARB_S_GBUAPCD_Keeler	36.48757	-117.871	28
Oakridge, OR	AirNow	41-039-2013	Met One BAM-1022 Mass Monitor w/ VSCC or TE-PM2.5C - Beta Attenuation	43.74435	-122.48	LRAPA-Oakridge 2	43.74431	-122.481	81
Oroville, CA	Airsis	lon_.121.546_lat_39.510_apcd.1029 BUTTE EBAM	E-BAM	39.510	-121.546	CARB_SMOKE_BCAQMD_OROVILLE	39.51039	-121.547	96
Tulelake, CA	Airsis	on_.121.479_lat_41.956_arb2.1010	E-BAM	41.956	-121.479	CARB_SMOKE_SCAPCD_TULELAKE_ELEMENTARY	41.95588	-121.480	84

3 Comparability of FRMs and FEMs

Table S3. Available FEM comparability assessments (13/17 FEM sites) (https://www.epa.gov/outdoor-air-quality-data/pm25-continuous-monitor-comparability-assessments, last access January 21, 2022). Assessments look at all data available for comparison from the past 3 years at 24-hr averages. The approximate maximum FRM $PM_{2.5}$ concentration was read off the generated scatter plots. Shaded cells did not meet the targets for regulatory monitors (slope=0.9 to 1.1, Intercept=-2 to 2).

City	State	County	AQS	monitor	Y=cont., x=FRM			Cont/ FRM	FRM $PM_{2.5}$ ($\mu g/m^3$)		Most Recent Year
					slope	int	R	ratio	Mean	Approx. Max	
Appleton	Wisconsin		55-087-0009	BAM-1020	0.95	-0.26	0.95	0.91	6.5	27	2017
Bend	Oregon	Deschutes	41-017-0120	Radiance Research M903 With Heated Inlet - Nephelometry	0.87	0.44	0.97	0.95	5.1	39	2010
Bishop	California	Inyo	6-027-0002	Teledyne T640X at 16.67 LPM - Broadband spectroscopy	1.65	-2.10	0.99	1.34	6.8	120	2021
Boise	Idaho	Ada	16-001-0010	Met-One BAM-1020 W/PM2.5 SCC - Beta Attenuation	0.99	1.26	0.97	1.16	7.5	85	2021
Cedar Rapids	Iowa	Linn	19-113-0040	Met One BAM-1020 Mass Monitor w/VSCC - Beta Attenuation	0.88	0.16	0.94	0.96	8.2	32	2020
Cedar Rapids	Iowa	Linn	19-113-0040	Teledyne T640 at 5.0 LPM - Broadband spectroscopy	0.96	1.63	1.63	1.16	8.2	32	2022
Decatur	Georgia	DelKalb	13-089-0002	Teledyne T640X at 16.67 LPM - Broadband spectroscopy	1.00	1.07	0.95	1.13	8.1	37	2021
Denver	Colorado	Denver	8-031-0026	Teledyne T640 at 5.0 LPM - Broadband spectroscopy	1.20	0.08	0.96	1.21	7.4	43	2021
Edmond	Oklahoma	Oklahoma	40-109-1037	Teledyne T640 at 5.0 LPM - Broadband spectroscopy,	0.98	2.35	0.89	1.30	7.3	33	2019
Edmond	Oklahoma	Oklahoma	40-109-1037	Teledyne T640X at 16.67 LPM - Broadband spectroscopy	1.26	1.78	0.88	1.52	6.8	24	2022
Keeler	California	Inyo	6-027-1003	Thermo Scientific TEOM 1400 FDMS or 1405 8500C FDMS w/VSCC - FDMS Gravimetric	1.28	0.39	1.00	1.34	6.7	115	2021
Mammoth	California	Mono	6-051-0001	Teledyne T640X at 16.67 LPM - Broadband spectroscopy	1.95	-4.10	0.99	1.65	13.7	310	2021
Oakridge	Oregon	Lane	41-039-2013	Met One BAM-1022 Mass Monitor w/ VSCC or TE-PM2.5C - Beta Attenuation	0.95	0.05	1.00	0.96	15.6	580	2020

Phoenix	Arizona	Maricopa	04-013-0019	Thermo Scientific TEOM 1405-DF Dichotomous FDMS - FDMS Gravimetric	1.02	0.07	0.98	1.02	8.6	32	2021
Sarasota	Florida	Sarasota	12-115-0013	Teledyne T640 at 5.0 LPM - Broadband spectroscopy	0.82	1.49	0.96	1.05	6.5	19	2018
Wilmington	Delaware	New Castle	10-003-2004	Teledyne T640 at 5.0 LPM - Broadband spectroscopy	0.97	1.19	0.96	1.13	7.6	32	2020

Table S 4. Unavailable FEM comparisons (https://www.epa.gov/outdoor-air-quality-data/pm25-continuous-monitor-comparability-assessments, last access January 21, 2022).

City	State	County	AQS	Monitor	Notes
Appleton	Wisconsin		55-087-0009	Teledyne T640 at 5.0 LPM - Broadband spectroscopy	No report for this monitor
Atascadero	California	San Luis Obispo	6-79-8002	Met One BAM-1020 Mass Monitor w/VSCC - Beta Attenuation	Site not listed
Topeka	Kansas	Shawnee	20-177-0013	Teledyne T640 at 5.0 LPM - Broadband spectroscopy	No reports
Marysville	Washington	Snohomish	53-061-1007	Thermo Scientific 1405-F FDMS w/VSCC - FDMS Gravimetric<br>Met One BAM-1020 Mass Monitor w/VSCC - Beta Attenuation	No report for either monitor
Missoula	Montana	Missoula	30-063-0024	Met One BAM-1020 Mass Monitor w/VSCC - Beta Attenuation	County not listed
RTP	North Carolina	Durham	37-063-0099	Teledyne T640X at 16.67 LPM - Broadband spectroscopy	Site not listed (not a regulatory site)

To better understand over which concentration range the T640s provide accurate data, 3 years of 24-hr averaged data were downloaded from AQS (2019-2021, download: January 2022). The T640 or T640x data were matched with FRM measurements and segmented regression (Muggeo 2008) was used to understand the change in bias over the full range of concentrations (24-hr average range 0-309 µg/m^3). In the segmented regression, the number of breakpoints was increased from 0-3 (Table S 3). The regression was run separately for T640 and T640x. We would expect the T640 and T640x to perform comparably, as to our knowledge the primary difference is the inlet utilized to meet FEM PM_{10} designation requirements. Using 2 breakpoints the T640 has a reasonable relationship with the FRM with a slope of 0.95 from 0-10 µg/m^3, and a slope of 1.13 from 10-30 with intercepts within 2 µg/m^3. However, above 30 µg/m^3 the accuracy is poor with a slope of 0.53 with an intercept of 18 µg/m^3. Results were similar for the T640x with a breakpoint near the limit of detection (0.52 µg/m^3) and good performance between the limit of detection and 35 µg/m^3 with a slope of 1.07. However, the accuracy is poor above 36 µg/m^3 with a slope of 1.99 with an intercept of -32 µg/m^3. The poor accuracy could be driven by extreme values experienced by the T640x but only looking at the piecewise regression from 0-80 µg/m^3 results in a similar breakpoint and worse slope and intercept above the top breakpoint. Due to the nonlinear performance of the T640 and T640x especially above 30-38 µg/m^3 we have excluded all high concentration (>35 µg/m^3) T640 data from this analysis. We have used 35 µg/m^3 for simplicity since it is the break between the moderate and the unhealthy for sensitive groups categories. This removes only 0.8% of the total dataset (removed: Decatur N=5, Denver N=230, Edmond N=69, Sarasota N=19, Topeka N=86, Wilmington N=27). No T640 or T640x nearby smoke-impacted monitors were used moving forward (Table S 4).

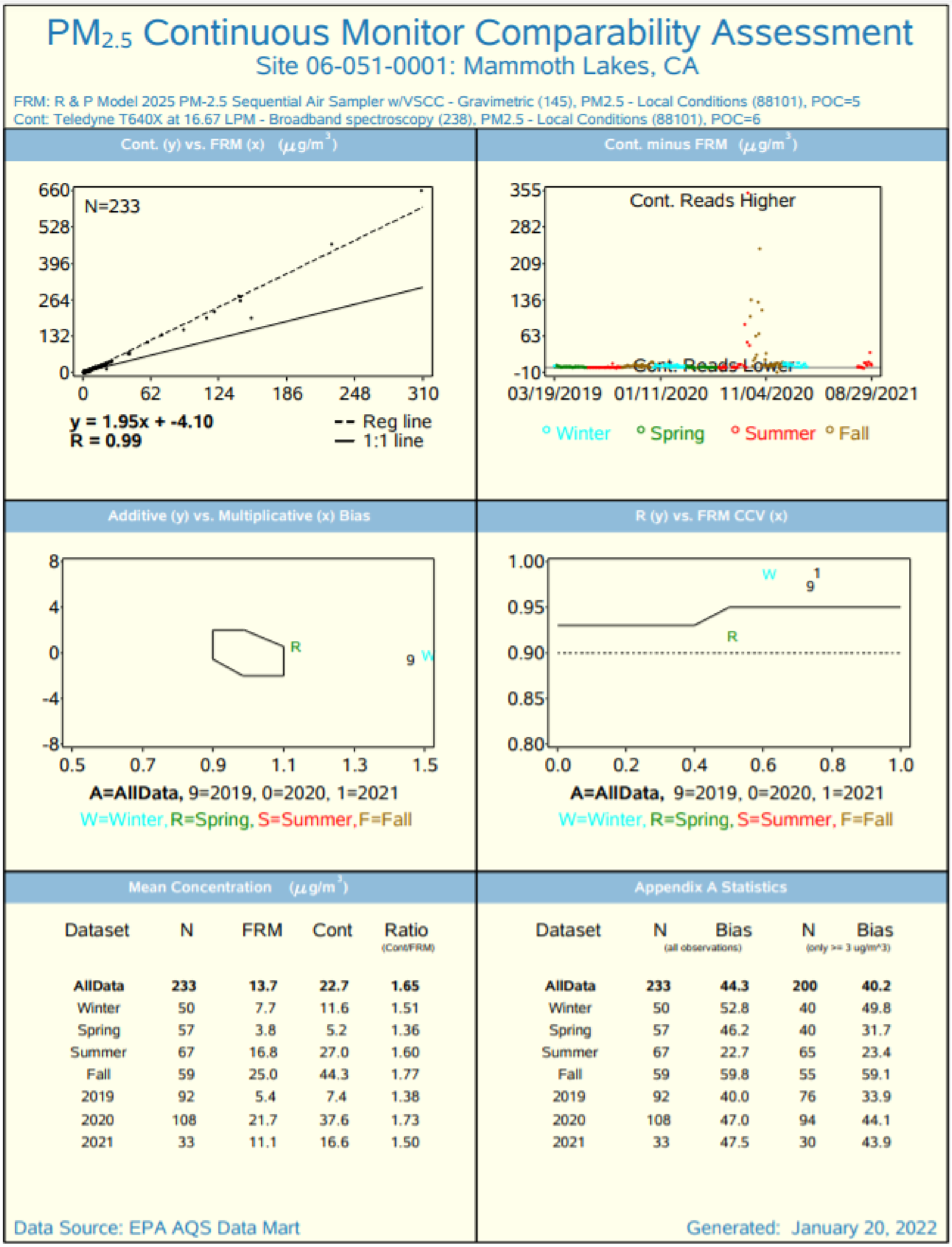

Dataset	N	FRM	Cont	Ratio (Cont/FRM)
**AllData**	**233**	**13.7**	**22.7**	**1.65**
Winter	50	7.7	11.6	1.51
Spring	57	3.8	5.2	1.36
Summer	67	16.8	27.0	1.60
Fall	59	25.0	44.3	1.77
2019	92	5.4	7.4	1.38
2020	108	21.7	37.6	1.73
2021	33	11.1	16.6	1.50

Dataset	N (all observations)	Bias	N (only >= 3 ug/m^3)	Bias
**AllData**	**233**	**44.3**	**200**	**40.2**
Winter	50	52.8	40	49.8
Spring	57	46.2	40	31.7
Summer	67	22.7	65	23.4
Fall	59	59.8	55	59.1
2019	92	40.0	76	33.9
2020	108	47.0	94	44.1
2021	33	47.5	30	43.9

Figure S 2**.** 24-hr averaged performance of T640x versus gravimetric measurements (https://www.epa.gov/outdoor-air-quality-data/pm25-continuous-monitor-comparability-assessments, last accessed January 20, 2022). Some points are outside the ranges on the plots.

Table S 5. We considered all 24-hr averaged data from T640s and T640x monitors compared to FRM for the past 3 years. (Data downloaded from https://aqs.epa.gov/aqsweb/airdata/download_files.html, access 1/21/2022). This analysis included 86 T640 sites and 62 T640x sites. We fit a segmented regression (using R package segmented –(Muggeo 2008)).

	T640 (N=23,447, FRM range: 0-77 μg/m³)						T640x (N=13,009, FRM range: 0-309 μg/m³)						T640x<80 (N= 12,997, FRM range: 0-72 μg/m³)					
# Break points (BP)	R^2_{adj}	RSE (μg/m³)	Slope or BP	Est	SE	Int (μg/m³)	R^2_{adj}	RSE (μg/m³)	Slope or BP	Est	SE	Int (μg/m³)	R^2_{adj}	RSE (μg/m³)	Slope or BP	Est	SE	Int (μg/m³)
0	0.87	1.90	S1	1.03	0.00	1.18	0.89	3.72	S1	1.52	0.00	-2.27	0.85	2.23	S1	1.11	0.00	0.70
			BP1	7.57	0.35		0.95	2.57	BP1	35.01	0.58		0.86	2.13	BP1	37.61	0.63	
			S1	0.94	0.01	1.63			S1	1.07	0.01	0.99			S1	1.07	0.00	1.01
1	0.87	1.89	S2	1.06	0.00	0.76			S2	1.99	0.01	-31.25			S2	2.31	0.05	-45.74
			BP1	9.89	0.25		0.95	2.56	BP1	0.52	0.06		0.86	2.12	BP1	0.52	0.05	
			BP2	29.94	0.43				BP2	35.62	0.61				BP2	37.61	0.56	
			S1	0.95	0.01	1.59			S1	-19.25	4.17	11.50			S1	-19.23	3.45	11.50
			S2	1.13	0.01	-0.22			S2	1.07	0.01	0.96			S2	1.07	0.00	0.99
2	0.87	1.86	S3	0.53	0.02	17.83			S3	1.99	0.01	-31.83			S3	2.31	0.05	-45.56
			BP1	0.80	0.08		0.95	2.56	BP1	0.55	0.07		0.86	2.12	BP1	3.49	1.38	
			BP2	10.08	0.27				BP2	9.95	0.68				BP2	8.86	0.56	
			BP3	30.66	0.46				BP3	37.13	0.75				BP3	38.05	0.72	
			S1	-6.60	1.34	7.55			S1	-16.44	4.16	10.93			S1	1.05	0.06	1.22
			S2	0.96	0.01	1.54			S2	1.01	0.01	1.31			S2	0.99	0.02	1.45
			S3	1.13	0.01	-0.20			S3	1.14	0.01	0.01			S3	1.12	0.01	0.27
3	0.86	1.85	S4	0.51	0.02	18.90			S4	1.99	0.01	-31.62			S4	2.27	0.05	-43.58

Table S 6. Reference monitor types and IDs for nearby sensor T640/T640x pairs identified on the Fire and Smoke map. These were not used in the analysis due to the high bias at high concentration.

City	Source	ID	Monitor type	Latitude	Longitude	Name	Latitude	Longitude	distance (m)
Bishop, CA	AirNow	6-027-0002	Teledyne T640X at 16.67 LPM - Broadband spectroscopy	37.36068	-118.331	GBUAPCD_NCORE 2	37.36049 7	-118.332	91
Mammoth Lakes, CA	AirNow	6-051-0001	Teledyne T640X at 16.67 LPM - Broadband spectroscopy	37.64571	-118.967	MammothLakes	37.6254	-118.981	2571

4 NowCast

AirNow's Air Quality Index (AQI) gives a daily measure of air quality conditions and associated health risk. While the AQI provides individuals with a 24-hr average measure of air quality, EPA has devised the NowCast as a means for reporting changes in air quality on higher time-resolution levels. The NowCast results in hourly PM concentration values computed via a 12-hour window of hourly PM average values. Hourly PM values are weighted based on how rapidly the concentration levels change within the 12-hour window; a high rate of change results in higher weighting of concentration values recorded for the most recent hours.

NowCast Calculation

The NowCast for a selected (current) hour is computed via the following steps:

1. Select a 12-hour window of PM measurements whereby the oldest hour in the selection is 11 hours preceding the current hour and the most recent (current) hour is the hour for which the NowCast is computed. At least two of the last three hours (including the current hour) must have concentration values to compute the NowCast.
2. From this 12-hour window, select the maximum and minimum PM concentrations present. Compute the range by subtracting the minimum from the maximum.
3. Normalize the range by dividing by the maximum concentration value. This gives a measure of the rate of change of PM values within the 12-hour window.
4. Compute the weight factor by subtracting the normalized rate of change from 1.
5. If the weight factor is less than 0.5, round the value up to 0.5. The weight factor must fall within the range 0.5 to 1.0.
6. Multiply each hour in the 12-hour window by the weight factor raised to the power of the number of hours ago that the value was recorded. The most recent (current) hour in the series is raised to the zeroth power and the oldest hour is raised to the 11th power.
7. Sum the weighted PM values computed in the previous step for each hour in the 12-hour window.
8. In a similar method to steps 6 and 7, compute the sum of the weight factor raised to the 0th through 11th power. This sum includes 12 terms, whereby the power of each term corresponds to the number of hours ago that a concentration value was recoded.
9. Divide the weighted concentration sum calculated in step 7 by the sum determined in step 8. The result is the NowCast for the zeroth (current) hour in the 12-hour window.

Table S 7. Air Quality Index (AQI) breakpoints used to convert $PM_{2.5}$ NowCast concentration into AQI (EPA 2021).

AQI Category	Low AQI	High AQI	Low Breakpoint (μg/m^3)	High Breakpoint (μg/m^3)
Good	0	50	0	12
Moderate	51	100	12.1	35.4
Unhealthy for sensitive groups (UHSG)	101	150	35.5	55.4
Unhealthy	151	200	55.5	150.4
Very unhealthy (VUH)	201	300	150.5	250.4
Hazardous	301	400	250.5	350.4
Hazardous	401	500	350.5	500.4
Hazardous	501	999	500.5	99999.9

5 Additional figures and tables

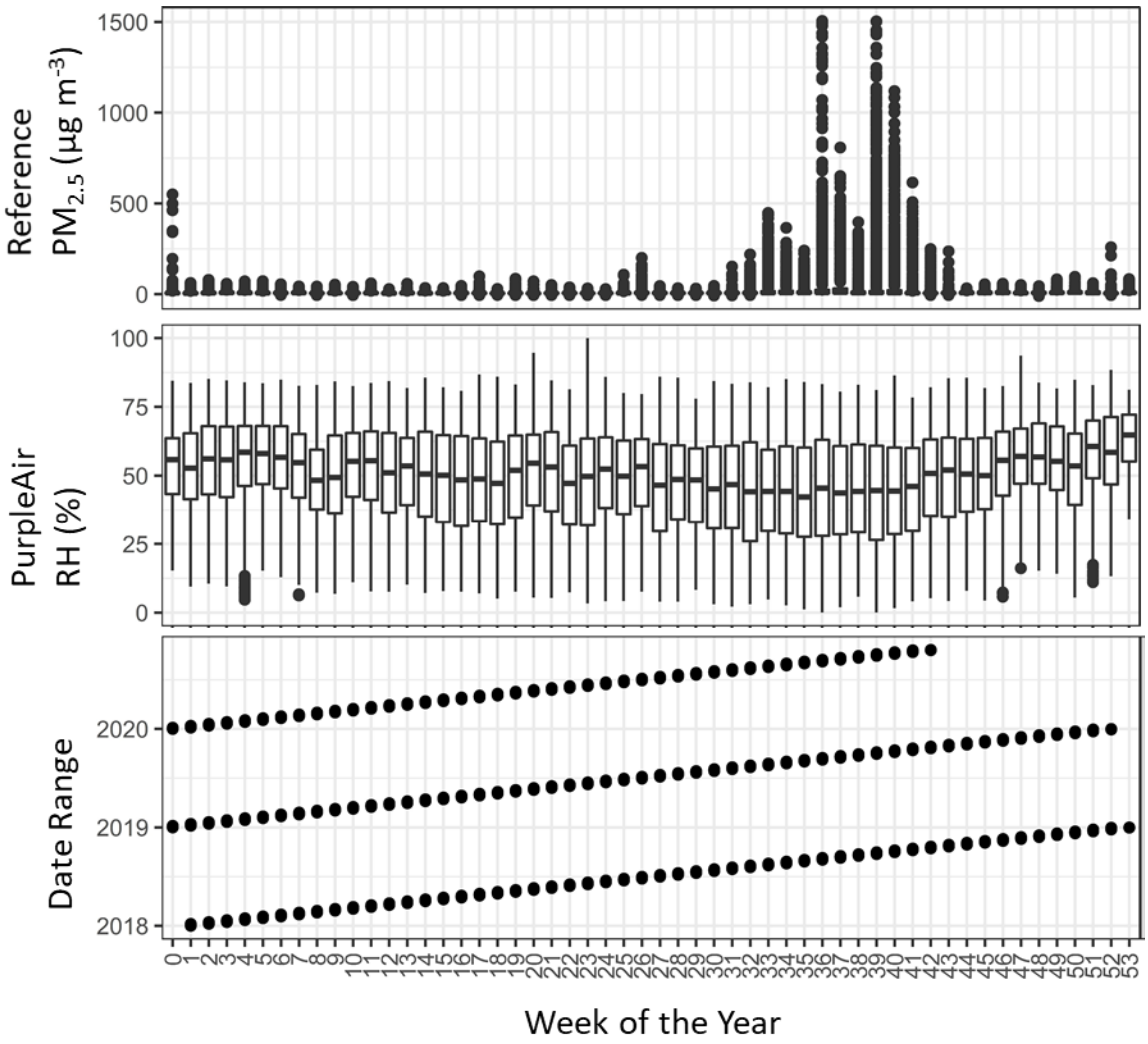

Figure S 3. Summary of distribution of RH and $PM_{2.5}$ by week of the year (withholding groups).

Table S 8. Comparison of error by correction equation A-D (from **Table 3**) binned by concentration range surrounding each air quality index breakpoint, the Cal/OSHA limit to put on a respirator (500 µg/m³), low concentration data (0-10 µg/m³), and extremely high concentrations (>600 µg/m³). Error is summarized by normalized mean bias error (NMBE), normalized mean absolute error (NMAE), and normalized root mean squared error (NRMSE). Grey shaded rows have an absolute NMBE greater than 10%.

Model	AQI breakpoint	Range $PM_{2.5}$ (µg/m³)	N	Leave one site out				Leave one week out			
				NMBE	NRMSE	RMSE	NMAE	NMBE	NRMSE	RMSE	NMAE
A-U.S.-wide	Below (Good)	0-10	90960	-5%	54%	3	40%	No withholding since preexisting equation			
	Good→Moderate	10-14	15205	-10%	31%	4	23%				
	Moderate→UHSG	28-42	2196	4%	27%	9	20%				
	UHSG→Unhealthy	44-66	1291	9%	22%	12	17%				
	Unhealthy→VUH	120-180	503	2%	12%	18	10%				
	VUH→Hazardous	200-300	475	-6%	12%	29	9%				
	Cal/OSHA	400-600	230	-24%	25%	123	24%				
	Beyond	600+	189	-41%	45%	392	41%				
B-Quadratic	Below (Good)	0-10	90960	16%	51%	3	35%	15%	50%	3	34%
	Good→Moderate	10-14	15205	-10%	27%	3	21%	-11%	27%	3	21%
	Moderate→UHSG	28-42	2196	-12%	26%	9	20%	-12%	25%	9	19%
	UHSG→Unhealthy	44-66	1291	-7%	20%	11	15%	-7%	19%	11	15%
	Unhealthy→VUH	120-180	503	1%	15%	22	12%	0%	15%	22	11%
	VUH→Hazardous	200-300	475	5%	17%	42	14%	6%	16%	41	13%
	Cal/OSHA	400-600	230	0%	16%	76	12%	1%	18%	85	13%
	Beyond	600+	189	-8%	23%	201	18%	-7%	25%	216	19%
C-Cubic	Below (Good)	0-10	90960	19%	51%	3	36%	18%	50%	3	35%
	Good→Moderate	10-14	15205	-12%	26%	3	21%	-12%	26%	3	21%
	Moderate→UHSG	28-42	2196	-15%	26%	9	21%	-15%	27%	9	21%
	UHSG→Unhealthy	44-66	1291	-10%	21%	11	16%	-10%	20%	11	16%
	Unhealthy→VUH	120-180	503	0%	15%	22	12%	-1%	15%	22	11%
	VUH→Hazardous	200-300	475	5%	17%	43	14%	5%	16%	40	13%
	Cal/OSHA	400-600	230	0%	17%	80	13%	1%	18%	85	13%
	Beyond	600+	189	-4%	32%	273	23%	-2%	33%	290	23%
D-Quadratic+RH	Below (Good)	0-10	90960	16%	50%	3	34%	15%	49%	3	33%
	Good→Moderate	10-14	15205	-11%	26%	3	21%	-11%	26%	3	20%
	Moderate→UHSG	28-42	2196	-11%	25%	9	19%	-11%	25%	8	19%
	UHSG→Unhealthy	44-66	1291	-6%	19%	11	15%	-6%	19%	10	14%
	Unhealthy→VUH	120-180	503	1%	15%	22	12%	0%	15%	21	11%
	VUH→Hazardous	200-300	475	5%	17%	42	14%	6%	16%	41	13%
	Cal/OSHA	400-600	230	0%	16%	77	12%	1%	18%	85	13%
	Beyond	600+	189	-8%	23%	201	18%	-7%	25%	217	19%

UHSG=Unhealthy sensitive groups, VUH=Very Unhealthy

Table S 9**.** Comparison of error by correction equation E, F (from Table 2) binned by concentration range surrounding each air quality index breakpoint, the CalOSHA limit to put on a respirator (500 μg/m^3), low concentration data (0-10 μg/m^3), and extremely high concentrations (>600 μg/m^3). Error is summarized by normalized mean bias error (NMBE), normalized mean absolute error (NMAE), and normalized root mean squared error (NRMSE). Grey shaded rows have an absolute NMBE greater than 10%.

Model	AQI breakpoint	Range $PM_{2.5}$ (μg/m^3)	N	Leave one site out				Leave one week out			
				NMBE	NRMSE	RMSE	NMAE	NMBE	NRMSE	RMSE	NMAE
E-Quadratic PM*RH	Below (Good)	0-10	90960	15%	51%	3	36%	14%	50%	3	35%
	Good→Moderate	10-14	15205	-10%	27%	3	21%	-10%	27%	3	20%
	Moderate→UHSG	28-42	2196	-11%	25%	9	20%	-11%	25%	8	19%
	UHSG→Unhealthy	44-66	1291	-8%	20%	11	16%	-8%	20%	11	15%
	Unhealthy→VUH	120-180	503	0%	16%	24	13%	0%	16%	23	13%
	VUH→Hazardous	200-300	475	5%	19%	47	15%	5%	17%	43	13%
	Cal/OSHA	400-600	230	0%	16%	76	11%	0%	17%	82	12%
	Beyond	600+	189	-8%	22%	191	17%	-7%	24%	208	18%
F- RH growth (Nilson)	Below (Good)	0-10	90960	-10%	67%	4	48%	-10%	66%	3	47%
	Good→Moderate	10-14	15205	8%	44%	5	32%	8%	43%	5	31%
	Moderate→UHSG	28-42	2196	46%	65%	22	52%	46%	65%	22	52%
	UHSG→Unhealthy	44-66	1291	63%	73%	40	65%	63%	73%	40	65%
	Unhealthy→VUH	120-180	503	54%	60%	88	55%	54%	60%	88	55%
	VUH→Hazardous	200-300	475	42%	47%	116	42%	41%	46%	115	42%
	Cal/OSHA	400-600	230	13%	23%	110	18%	13%	22%	108	18%
	Beyond	600+	189	-13%	25%	214	16%	-13%	25%	214	16%

UHSG=Unhealthy sensitive groups, VUH=Very Unhealthy

Table S 10. Error in the transition zone between the U.S.-wide correction and the quadradic fit at higher granularity (20 µg/m³ bins).

Reference Concentration range $PM_{2.5}$ (µg/m³)	Equation	N	U.S.-wide correction NMBE	Quad fit (LOSO)	Transition raw 570-611 (300 U.S.-wide to 400 quadratic) (LOSO)	Quad fit (LOBD)	Transition raw 570-611 (300 U.S.-wide to 400 quadratic) (LOBD)
0-200	1	67667	-3%	0%	-3%	0%	-3%
200-220	1	91	-2%	4%	-2%	4%	-2%
220-240	1	103	-6%	1%	-6%	2%	-6%
240-260	1	112	-5%	7%	-5%	7%	-5%
260-280	1	86	-7%	6%	-7%	7%	-7%
280-300	1	83	-8%	6%	-7%	7%	-7%
300-320	1	71	-11%	5%	-9%	6%	-9%
320-340	1	58	-11%	7%	-4%	8%	-3%
340-360	1	53	-11%	9%	1%	10%	1%
360-380	1	49	-17%	1%	-6%	3%	-5%
380-400	1	31	-18%	1%	-3%	3%	-2%
400-420	1	33	-19%	2%	-1%	4%	1%
>420	1	386	-35%	-5%	-6%	-4%	-4%

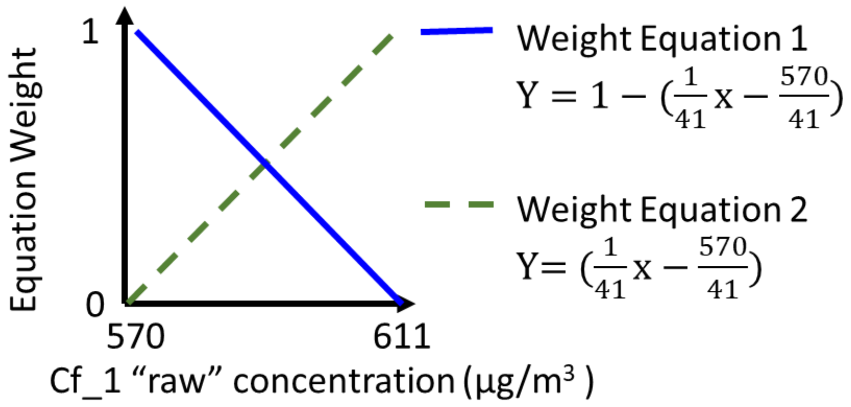

Figure S 4. Weighted transition equation to switch from the U.S.-wide correction at raw [cf=1] PurpleAir concentrations <570 µg/m³ to the quadradic equation at [cf=1] PurpleAir concentrations >611 µg/m³.

If all the Teledyne T640 and T640x data are excluded the NMBE is somewhat different although still acceptable (NMBE ≤ 10%) at each AQI breakpoint (Table S11). The NMBE in the 0-10 μg/m^3 range is 12% suggesting that the PurpleAir may overestimate compared to the monitors other than T640 and T640x. The NMBE at the breakpoint to moderate is -3% without any of the T640 or T640x data compared to -10% with the T640 data included. These differences in results may be due in part to differences in measurement methods but also due to the parts of the country that are operating monitors other than T640s and T640xs (most data from Atascadero, Cedar Rapids, Marysville, Phoenix). These parts of the country may be impacted by more dust and other particle types that scatter less light per mass. This highlights the importance of carefully selecting collocation sites and monitors.

Table S 11**.** 1-hr error at each AQI breakpoint using site withholding and a transition from the U.S.-wide correction to a quadradic fit from 300-400 μg/m^3 (raw [cf=1] 570-611 μg/m^3). Error is also shown without Teledyne T640 and T640x data included.

AQI breakpoint	Concentration Range (μg/m^3)	N	Selected fit	NMBE	RMSE	NRMSE	No T640 or T640x		
							N	NMBE	RMSE
Below	0-10	90960	U.S.-wide	-5%	3	54%	49095	12%	3
Moderate	10-14	15205	U.S.-wide	-10%	4	31%	6222	-3%	4
UHSG	28-42	2196	U.S.-wide	4%	9	27%	1840	7%	9
Unhealthy	44-66	1291	U.S.-wide	9%	12	22%	*		
Very Unhealthy	120-180	503	U.S.-wide	2%	18	12%			
Hazardous	200-300	475	U.S.-wide	-5%	31	12%			
Cal/OSHA	400-600	230	Quadradic	0%	89	18%			
Beyond	600+	189	Quadradic	-7%	216	25%			

*No T640 data >35 μg/m^3 included

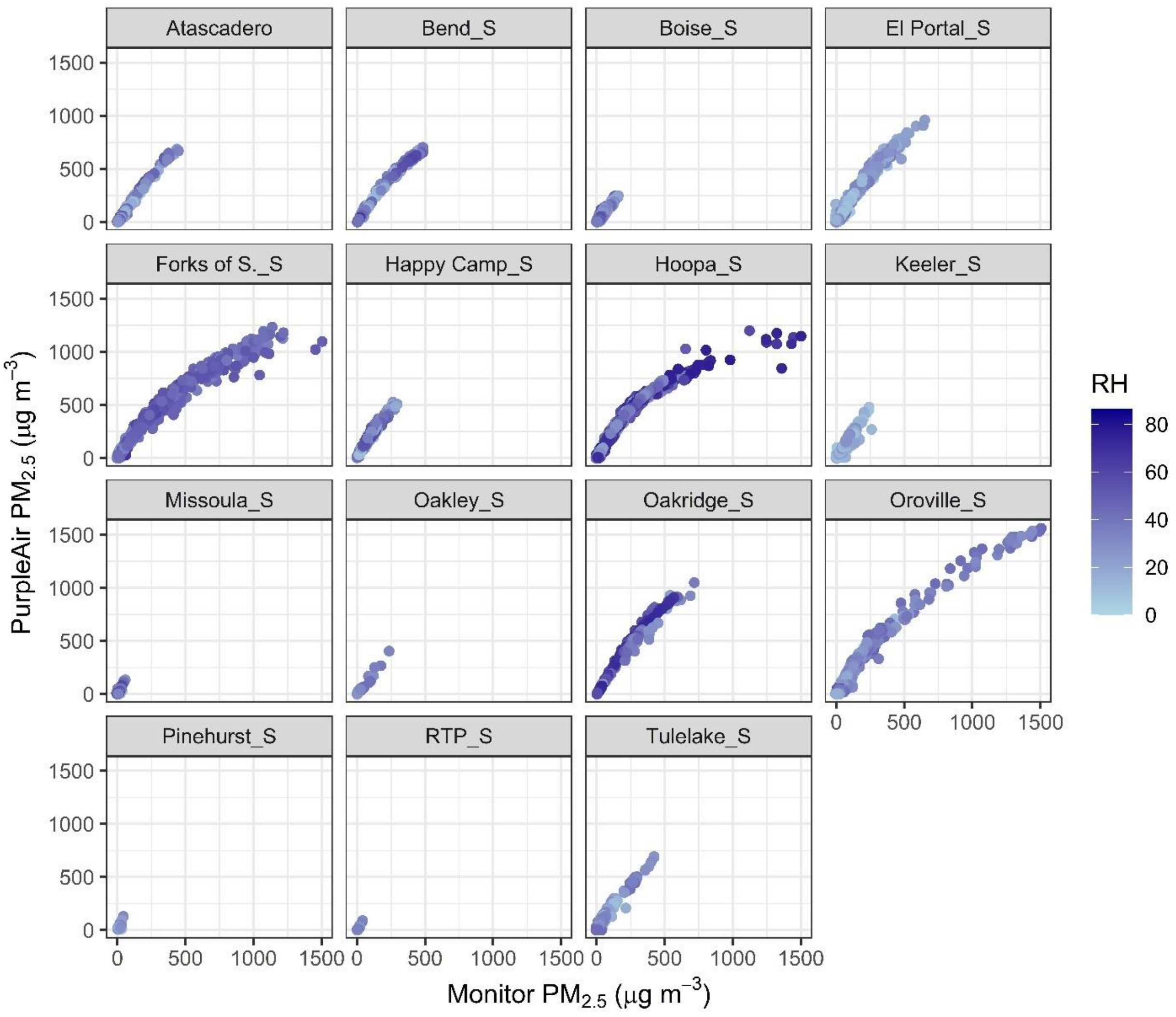

Figure S5**.** hourly averaged uncorrected PurpleAir [cf=1] data compared to the monitor $PM_{2.5}$ for each smoke-impacted site colored by PurpleAir RH.

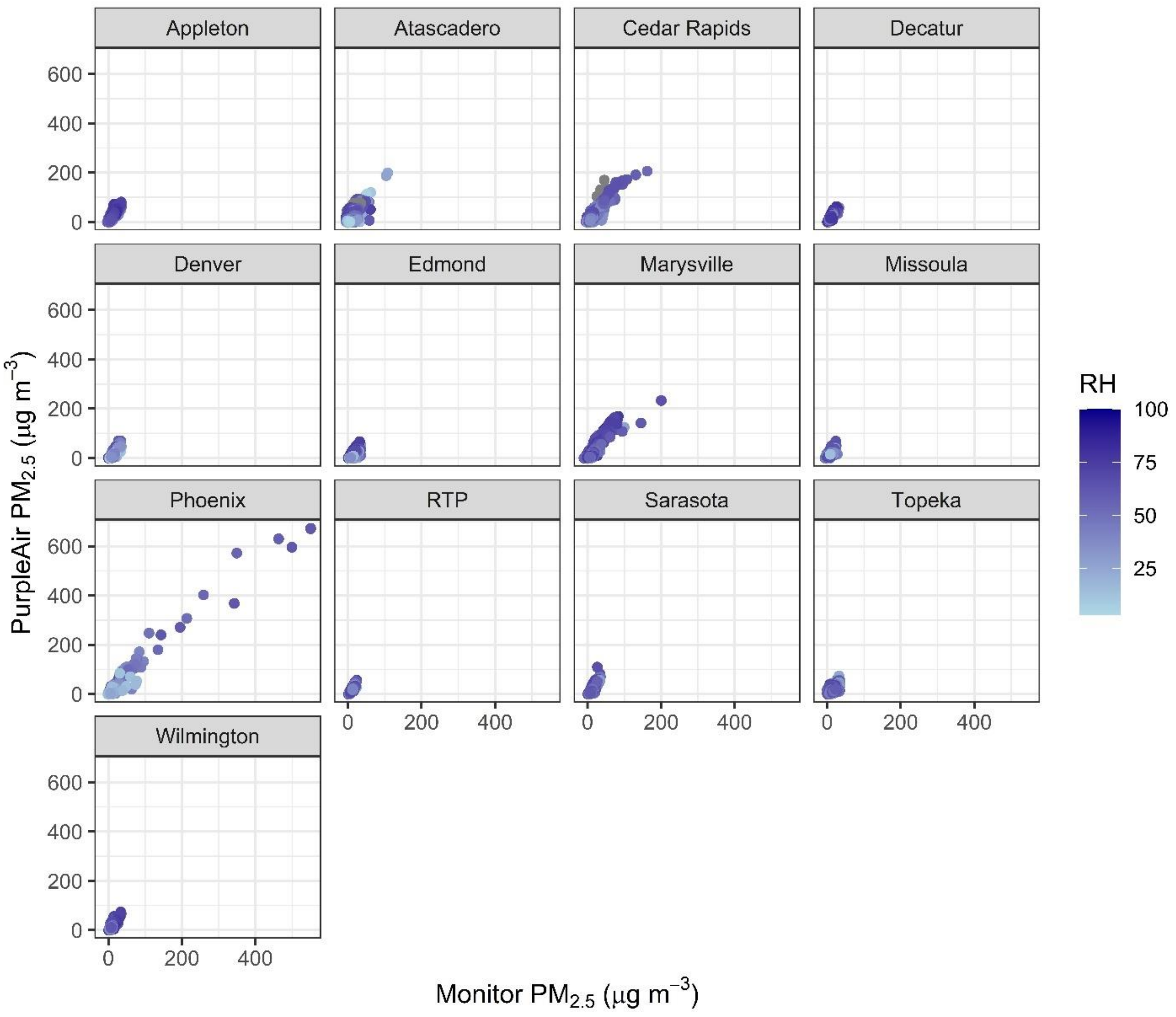

Figure S6**.** hourly averaged uncorrected PurpleAir [cf=1] data compared to the monitor $PM_{2.5}$ for each ambient site colored by PurpleAir RH.

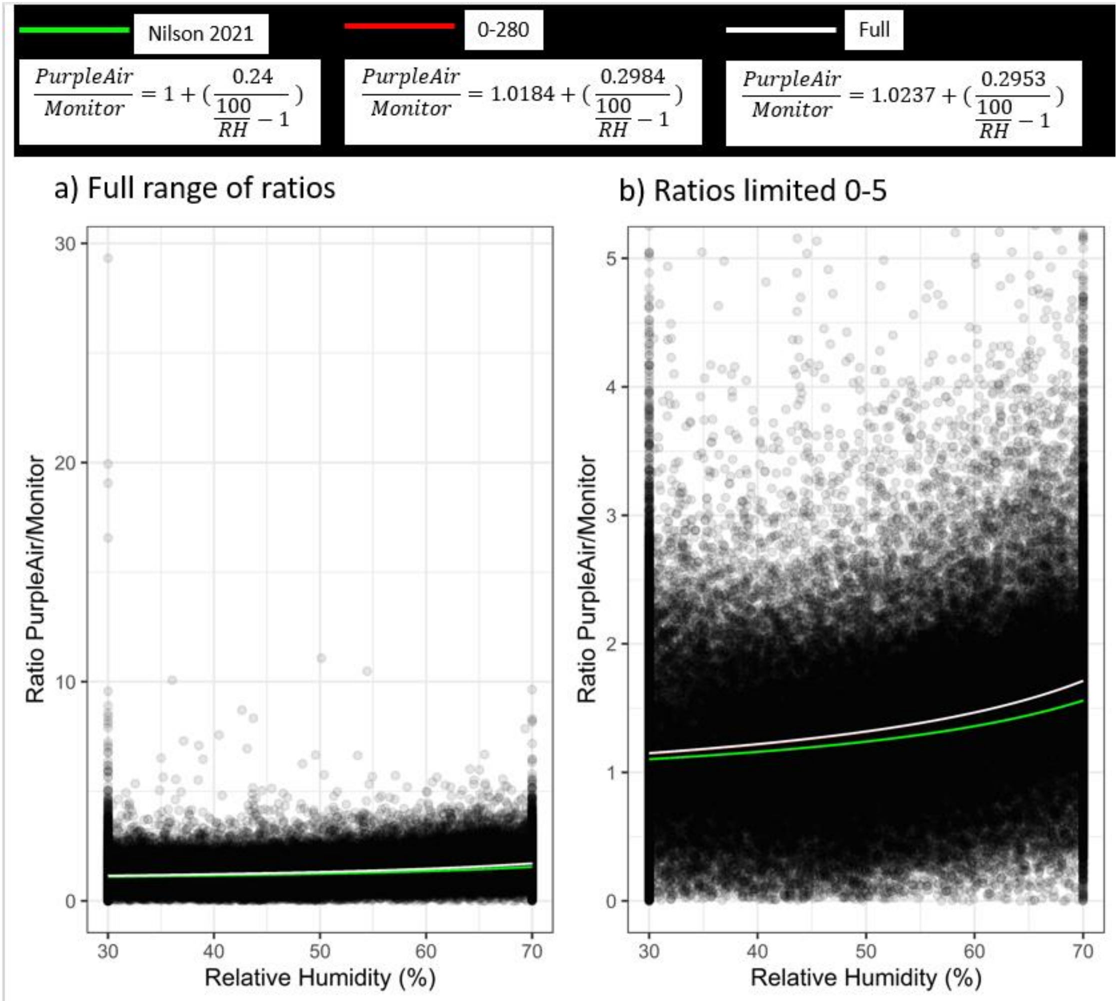

Figure S7. Hourly ratio of PurpleAir to Monitor (when the monitor is > 5 µg/m^3) versus relative humidity with fits based on Nilson (Nilson, Jackson et al. 2022). The equation from the Nilson paper is compared to equations generated from this dataset using the full range of data and only data 0-280 µg/m^3. The equations generated based on this dataset for the full range and from 0-280 µg/m^3 are similar with the white line almost completely covering the red line.

6 Detailed discussion of EPA performance targets

Ambient 24-hr

Both withholding methods resulted in similar performance with values typically only varying by a few µg/m^3 or percent (leave one sensor out: Table S12, Table S13 , leave one week out: Table S 14). At 24-hr averages, all but one site (Sarasota, FL) meet the targets. In Sarasota, the R^2 is <0.7 likely due to the more limited range of concentrations after averaging (0-20 µg/m^3). At 24-hr averages, six of the thirteen typical ambient sites did not reach 25 µg/m^3 which is recommended in the performance targets to ensure an evaluation over a wide enough range of concentrations. Note that these collocations occurred for 2 months to more than 2 years while the performance targets recommend 30-day evaluations. Shorter 30-day evaluations would be less likely to meet the 25 µg/m^3 target maximum concentration and could result in different performance metrics. At 24-hr averages, the typical ambient sites often had strong linearity (R^2 = 0.66 to 0.96), low bias (slope = 0.69 to 1.14, intercept = -1.99 to 2.12) and low error (RMSE = 2 to 6 µg/m^3).

Ambient 1-hr

While the performance targets recommend evaluating sensor performance at 24-hr averages, we also need to understand the performance at higher time resolution including hourly averages since air sensor data are often used at these higher time resolutions. The hourly data at all but one typical ambient site, Atascadero, meets all the performance target metrics (**Figure S5**). At hourly averages, the typical ambient sites often had strong linearity (R^2 = 0.53 to 0.96) though in some sites the R^2 is somewhat lower at 1-hr than at 24-hr averages. The R^2 in Atascadero shows the largest decrease from 0.80 at 24-hr averages to 0.53 at 1-hr averages. This may be due to noise in the sensor and comparison instrument, changes in the particle properties across hours, or other issues with the sensor not identified by the AB channel comparison. Sites typically have low bias (slope = 0.69 to 1.14, intercept = -1.99 to 2.12) and low error (RMSE = 2 to 6 μg/m^3). Due to the low average concentrations in these datasets, the percent error for many of these sensors is high (NRMSE = 21% to 84%), but since the performance targets specify sensors should meet either the NRMSE or the RMSE, all ambient sites meet the error target. These performance metrics were primarily designed with typical ambient concentration evaluations in mind. The NRMSE was added to the RMSE specifically for times where the average concentrations may be unusually high, including during wildfire smoke situations.

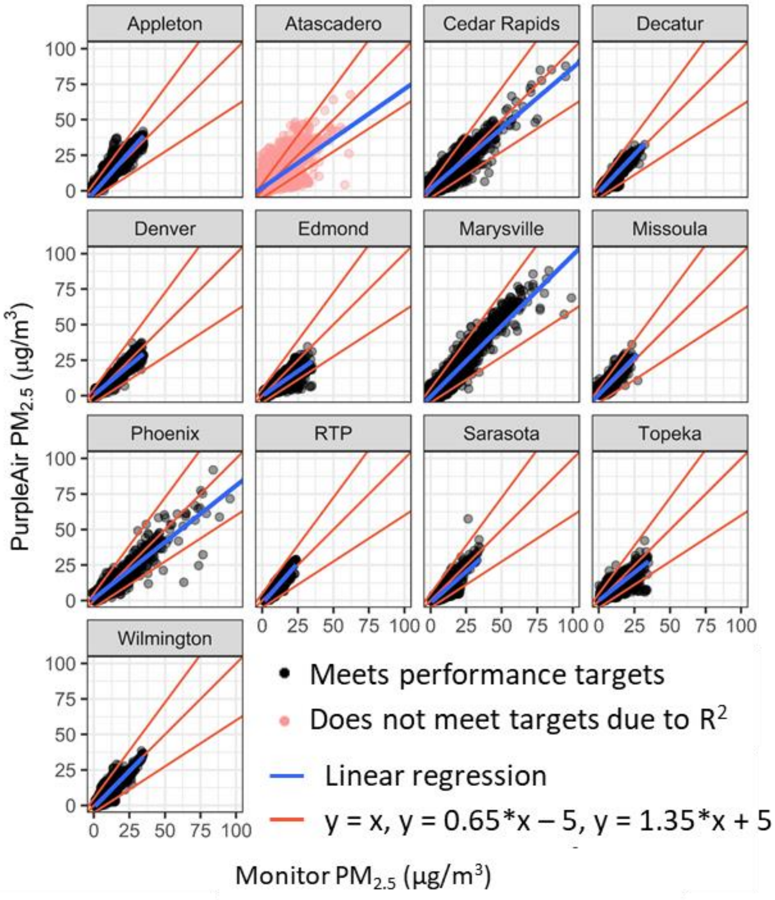

Figure S8. Hourly corrected PurpleAir $PM_{2.5}$ versus monitor data at each typical ambient site. Data were corrected using leave one week out withholding. Red lines indicate y=x, y=1.35x+5 and y=0.65x-5 with the upper and lower lines being the range of acceptable bias in the U.S. EPA performance targets and the blue line is linear regression. Plots are colored by whether they meet the performance targets at 1-hr averages.

Smoke 24-hr

Eight of fourteen smoke-impacted sensors did not meet the performance targets (Figure S6). The Oakley sensor is not included in the 24-hr averaged regression results since the dataset only contained one 24-hr average to compare.

The Missoula sensor has a high bias slightly outside the performance targets (slope=1.38). Five of the smoke-impacted sensors have intercepts > ±5 µg/m^3, which may not be of large concern because of the degree of uncertainty in the intercept that is likely with such a wide range of concentrations represented in the dataset Pinehurst is still the only site that does not meet the R^2 criteria at 24-hr averages potentially due in part to the limited range of concentrations (max=17 µg/m^3). Three of the smoke-impacted sites did not meet the RMSE/NRMSE targets. However, two of these sites are very close with errors of 31% and 32%. Many smoke-impacted sensors did not meet the performance targets; however, many are close still showing that they have utility during smoke-impacted times when other more accurate data are not available.

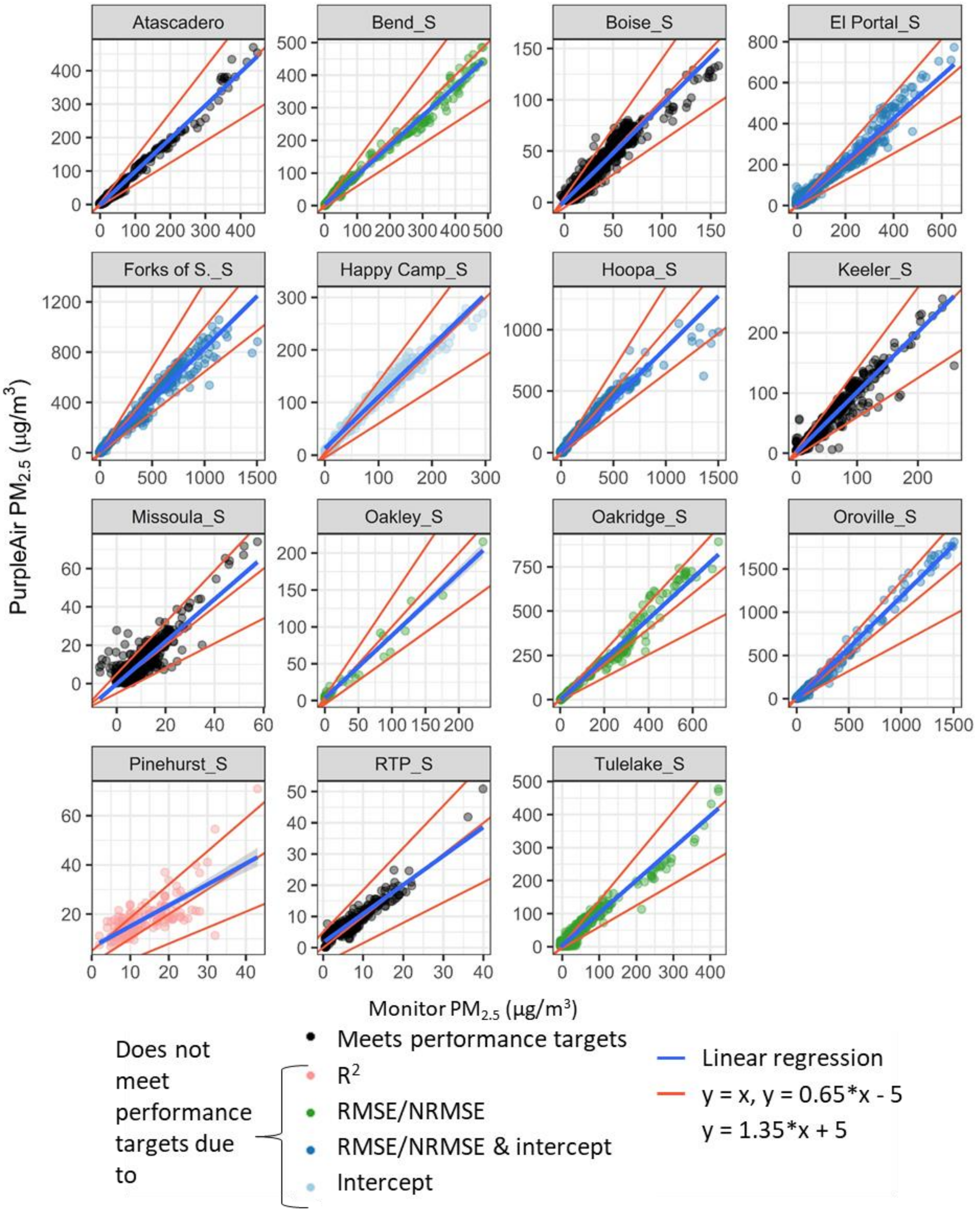

Figure S9. Hourly corrected PurpleAir $PM_{2.5}$ versus monitor data in each smoke-impacted site. Data were corrected using leave one week out withholding. Plots are colored based on meeting the performance targets

at 1-hr averages or the parameter that does not meet the performance targets. Red lines indicate y=x, y=1.35x+5 and y=0.65x-5 with the upper and lower lines being the range of acceptable bias in the U.S. EPA performance targets. The blue line is the linear regression.

Smoke 1-hr

Only five of ten smoke-impacted sensors met all the performance targets at 1-hr averages. Six sensors have intercepts outside the ± 5 μg/m^3 range (intercepts = -7.07 to 12.59). However, this may not be of large concern because of wide concentration ranges experienced and the impact on intercept. Only one site, Pinehurst, does not meet the linearity criteria (R^2 = 0.54) although a time series plot reveals many similar trends compared to the monitor (Figure S7). The low R^2 may be due in part to the limited range of concentrations (0 to 42 μg/m^3), limited period of evaluation (157 hours, <1 week), and the comparison with a BAM1020 that has noisier hourly averages at lower concentrations. The other smoke-impacted sensors typically have higher linearity (R^2 = 0.76 to 0.99) which is typically higher than the ambient sensors (All smoke R^2 = 0.95, All ambient R^2 = 0.79), likely due to their wider concentration ranges. Eight smoke-impacted sensors did not meet the RMSE and NRMSE criteria. The RMSE and NRMSE suggest there may be more uncertainty in the hourly data during smoke impacts than during typical ambient conditions. However, the slope values (i.e., bias) within the desired range suggest that there is confidence in the longer-term averages (e.g., 24-hr, weekly, seasonal, annual) provided by these sensors. The performance targets are not a pass/fail certification but are a recommendation to encourage overall performance improvement of technology entering the market. Falling short of these targets does not mean that the data are unusable.

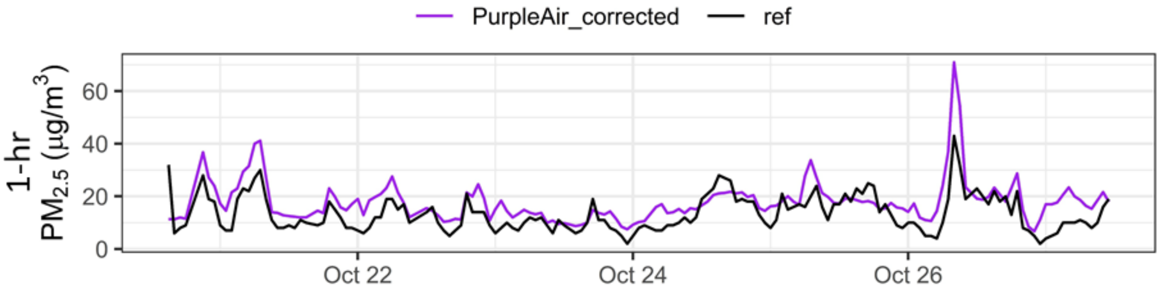

Figure S10. Pinehurst is the most poorly correlated of the smoke-impacted sensors. However, it still frequently shows similar trends. It is a short collocation which may contribute to the poor agreement.

It is important to note that not all recommendations in the performance targets were followed in this evaluation. Many of the smoke-impacted sites were selected as nearby monitor sensor pairs on the fire and smoke map and therefore may not be true collocations. Some of the scatter (RMSE) in the comparisons may be due to real concentration differences. In addition, the performance targets report recommends evaluating sensors in triplicates. Here, we have evaluated a single sensor at each site. However, past work with PurpleAir sensors has already established their high precision (especially using the duplicate Plantower sensors for quality control) and so this may be less of an issue for this sensor type (Feenstra, Papapostolou et al. 2019, Tryner, L'Orange et al. 2020, Wallace, Ott et al. 2020, Barkjohn, Gantt et al. 2021). Lastly not all sensors were evaluated against FEMs as recommended in the performance targets document (Duvall, Clements et al. 2021). However, this recommendation is less relevant for smoke monitoring where FEMs also have uncertainty since their designation involves field testing at typical ambient, not smoke-impacted, conditions.

Many of the slopes and intercepts vary between the 1-hr and 24-hr averaged datasets. Many of the sensors have higher slopes at 24-hr averages than at 1-hr averages (N=19/28, 68%) with six of these sensors having a difference in slope ≥0.10. The sensors with the largest increases in slope are Atascadero ambient (0.39 increase), Missoula smoke-impacted sensor (0.27 increase), the Missoula ambient sensor (0.17 increase), Marysville ambient (0.16), Cedar rapids ambient (0.11), and Pinehurst smoke (0.10 increase). Ordinary least squares regression minimizes the sum of the vertical

distances between each point and the regression line and passes through the mean of X and Y. If both the X and Y variables are noisy, the slope, as estimated by the ordinary least squares regression coefficient, will be biased low (Clarke and Van Gorder 2013). In addition, some of these sites had outliers where the sensor measured lower than the reference in the 1-hr averages that were less influential in the 24-hr averages (Figure S 6, Figure S 7). Only the RTP smoke-impacted datasets shows a decrease in slope ≥0.10 (0.12), likely due to two high concentration outliers that are less influential at 24-hr averages. While exploring the slope and intercept at 24-hr averages gives us more confidence in their values, it is important to note that we need to build and evaluate an extended correction on 1-hr averages to capture a wider range of $PM_{2.5}$ concentrations.

Table S 12. Ambient 24-hr and hourly average performance by sensor using site withholding. Shaded values did not meet EPA's performance targets for 24-hr averaged data. Where the number of hours (N), slope (m), intercept (b), R^2, root mean squared error (RMSE, µg/m^3), normalized root mean squared error (NRMSE), mean of the monitor, and max (µg/m^3) of the monitor are reported.

	24-hr averaged								1-hr averaged								Difference 24-hr to 1-hr			
City	N	m	b	R^2	RMSE	NRMSE	$PM_{2.5}$ mean	$PM_{2.5}$ max	N	m	b	R^2	RMSE	NRMSE	$PM_{2.5}$ mean	$PM_{2.5}$ max	m	R^2	RMSE	NRMSE
	≥30	1 ± 0.35	-5 ≤ b ≤5	≥0.70	≤ 7	≤30%	--	≥24	≥648	1 ± 0.35	-5 ≤ b ≤5	≥0.70	≤ 7	≤30%	--	≥24				
Appleton	357	1.16	-1.37	0.94	2	20%	8	29	8722	1.14	-1.28	0.91	2	28%	8	35	0.02	0.03	0	-8%
Atascadero	634	1.08	0.03	0.80	2	43%	5	34	15010	0.69	2.12	0.53	5	84%	5	108	0.39	0.27	-3	-41%
Cedar Rapids	806	0.96	0.67	0.78	2	28%	8	35	19507	0.85	1.51	0.75	3	41%	8	162	0.11	0.03	-1	-13%
Decatur	269	1.14	-1.69	0.92	1	14%	9	19	6868	1.06	-0.96	0.86	2	21%	9	32	0.08	0.06	-1	-7%
Denver	361	0.84	0.56	0.92	1	18%	8	22	8828	0.83	0.61	0.89	2	25%	8	35	0.01	0.03	-1	-7%
Edmond	286	0.71	-0.20	0.78	3	37%	9	25	8174	0.69	-0.16	0.76	4	42%	10	35	0.02	0.02	-1	-5%
Marysville	286	1.16	-0.99	0.94	2	27%	8	41	10515	1.00	-0.75	0.86	4	47%	8	200	0.16	0.08	-2	-20%
Missoula	70	1.24	-0.33	0.94	2	31%	6	15	1738	1.04	0.94	0.77	3	49%	6	27	0.20	0.17	-1	-18%
Phoenix	152	0.8	1.42	0.99	3	30%	10	149	3652	0.79	1.47	0.96	6	57%	10	550	0.01	0.03	-3	-27%
RTP	77	1.15	-2.23	0.80	2	21%	9	15	1921	1.13	-1.99	0.79	2	26%	9	24	0.02	0.01	0	-5%
Sarasota	380	0.84	-1.30	0.66	3	42%	7	20	9145	0.88	-1.66	0.70	3	45%	7	35	0.04	0.04	0	-3%
Topeka	326	0.8	0.06	0.82	2	27%	8	23	8281	0.79	0.16	0.78	3	34%	9	35	0.01	0.04	-1	-7%
Wilmington	231	1.02	-1.33	0.85	2	25%	8	30	5705	1.02	-1.34	0.85	2	29%	8	35	0.00	0.00	0	-4%
All ambient	4235	0.93	-0.02	0.82	2	30%	8	149	108066	0.93	1.16	0.79	3	43%	8	550	0.00	0.03	-1	-13%

Table S 13. Smoke-impacted 24-hr and hourly averaged performance by sensor using site withholding. Shaded values did not meet EPA's performance targets for 24-hr averaged data. Where the number of hours (N), slope (m), intercept (b), R^2, root mean squared error (RMSE), normalized root mean squared error (NRMSE), mean of the monitor, and max of the monitor are reported.

City	24-hr averaged								1-hr averaged								Difference 24-hr to 1-hr			
	N	m	b	R^2	RMSE	NRMSE	$PM_{2.5}$ mean	max	N	m	b	R^2	RMSE	NRMSE	$PM_{2.5}$ mean	max	m	R^2	RMSE	NRMSE
	≥30	1 ± 0.35	-5 ≤ b ≤5	≥0.70	≤ 7	≤30%	--	≥24	≥648	1 ± 0.35	-5 ≤ b ≤5	≥0.70	≤ 7	≤30%	--	≥24				
Atascadero	78	0.99	-0.91	1	3	11%	24	242	1850	0.99	-0.99	0.99	5	23%	23	448	0.00	0.01	-2	-12%
Bend	70	0.89	2.72	1	5	26%	20	252	1758	0.9	2.6	0.99	10	33%	30	485	-0.01	0.01	-5	-7%
Boise	72	0.96	0.67	0.97	3	16%	21	120	1753	0.94	1.04	0.94	5	26%	21	158	0.02	0.03	-2	-10%
El Portal	56	1.03	7.27	0.99	14	19%	73	382	1279	1.04	6.89	0.96	23	31%	73	651	-0.01	0.03	-9	-12%
Forks of Salmon	32	0.83	6.7	0.99	61	22%	273	816	1199	0.82	10.97	0.97	61	32%	191	1504	0.01	0.02	0	-10%
Happy Camp	14	0.98	13.44	0.98	13	14%	87	157	348	0.98	12.59	0.97	16	18%	86	295	0.00	0.01	-3	-4%
Hoopa	64	0.86	9.59	0.99	29	32%	90	887	1632	0.84	10.43	0.96	39	50%	80	1502	0.02	0.03	-10	-18%
Keeler	78	1.04	0.36	0.99	4	14%	26	115	1876	1	1.49	0.95	8	29%	27	260	0.04	0.04	-4	-15%
Missoula	46	1.38	-2.42	0.97	2	24%	8	20	1152	1.11	-0.13	0.76	4	48%	8	57	0.27	0.21	-2	-24%
Oakley	1	--	--	--	--	--	17	17	48	0.85	3.38	0.97	11	40%	27	236	--	--	--	--
Oakridge	77	1.12	-1.87	0.99	14	39%	35	489	1865	1.14	-2.15	0.98	20	55%	36	717	-0.02	0.01	-6	-16%
Oroville	40	1.16	-5.29	1	34	31%	111	852	1016	1.19	-7.07	0.99	49	48%	103	1506	-0.03	0.01	-15	-17%
Pinehurst	6	0.95	5	0.61	5	35%	13	17	157	0.85	6.54	0.54	7	53%	13	43	0.10	0.07	-2	-18%
RTP	17	0.81	2.39	0.88	2	25%	7	13	429	0.93	1.62	0.88	2	31%	7	40	-0.12	0.00	0	-6%
Tulelake	78	1.02	3.22	0.98	6	27%	23	213	1720	0.98	4.17	0.95	10	45%	23	422	0.04	0.03	-4	-18%
All smoke*	729	0.94	3.53	0.97	18	38%	50	887	18082	1.01	-1.1	0.95	25	51%	49	18082	-0.07	0.02	-7	-13%
ALL**	4964	0.95	0.3	0.97	7	54%	14	887	126148	1	0.46	0.96	10	73%	14	1506	-0.05	0.01	-3	-19%

*All smoke is a summary of all data shown in this table

**ALL is a summary of all smoke and ambient data

Table S 14. hourly and 24-hour averaged performance using leave one week out withholding. Grey shaded values did not meet EPA's performance targets for 24-hr data.

	24-hr Performance						Monitor $PM_{2.5}$		1-hr Performance						Monitor $PM_{2.5}$	
city	N	m	b	R^2	RMSE	NRMSE	Mean	Max	N	m	b	R^2	RMSE	NRMSE	Mean	Max
Target:	≥30	1 ± 0.35	-5 ≤ b ≤5	≤0.70	≤ 7	≤30%	--	≥25	≥720	1 ± 0.35	-5 ≤ b ≤5	≤0.70	≤ 7	≤30%	--	≥25
Appleton	357	1.16	-1	0.94	2	20%	8	29	8722	1.14	-1	0.91	2	28%	8	35
Atascadero	634	1.08	0	0.80	2	43%	5	34	15010	0.69	2	0.53	5	84%	5	108
Cedar Rapids	806	0.96	1	0.78	2	28%	8	35	19507	0.85	2	0.75	3	41%	8	162
Decatur	269	1.14	-2	0.92	1	14%	9	19	6868	1.06	-1	0.86	2	21%	9	32
Denver	361	0.84	1	0.92	1	18%	8	22	8828	0.83	1	0.89	2	25%	8	35
Edmond	286	0.71	0	0.78	3	37%	9	25	8174	0.69	0	0.76	4	42%	10	35
Marysville	286	1.16	-1	0.94	2	27%	8	41	10515	1.00	-1	0.86	4	47%	8	200
Missoula	70	1.24	0	0.94	2	31%	6	15	1738	1.04	1	0.77	3	49%	6	27
Phoenix	152	0.8	1	0.99	3	29%	10	149	3652	0.80	1	0.96	6	57%	10	550
RTP	77	1.15	-2	0.8	2	21%	9	15	1921	1.13	-2	0.79	2	26%	9	24
Sarasota	380	0.84	-1	0.66	3	42%	7	20	9145	0.88	-2	0.70	3	45%	7	35
Topeka	326	0.8	0	0.82	2	27%	8	23	8281	0.79	0	0.78	3	34%	9	35
Wilmington	231	1.02	-1	0.85	2	25%	8	30	5705	1.02	-1	0.85	2	29%	8	35
All ambient	4235	0.93	0	0.82	2	30%	8	149	108066	0.93	1	0.79	3	43%	8	550
Atascadero_S	78	0.99	-1	1.00	3	11%	24	242	1850	0.99	-1	0.99	6	23%	23	448
Bend_S	70	0.9	3	1.00	5	24%	20	252	1758	0.91	3	0.99	9	31%	30	485
Boise_S	72	0.96	1	0.97	3	16%	21	120	1753	0.94	1	0.94	5	26%	21	158
El Portal_S	56	1.04	7	0.99	14	20%	73	382	1279	1.05	7	0.96	23	31%	73	651
Forks of S._S	32	0.83	7	0.99	61	22%	273	816	1199	0.82	11	0.97	61	32%	191	1504
Happy Camp_S	14	0.98	13	0.98	13	14%	87	157	348	0.98	13	0.97	16	18%	86	295
Hoopa_S	64	0.86	10	0.99	29	32%	90	887	1632	0.84	10	0.96	39	49%	80	1502
Keeler_S	78	1.04	0	0.99	4	14%	26	115	1876	1.00	1	0.95	8	29%	27	260
Missoula_S	46	1.38	-2	0.97	2	24%	8	20	1152	1.11	0	0.76	4	48%	8	57
Oakley_S	1	--	--	--	--	--	--		48	0.85	3	0.97	11	40%	27	236
Oakridge_S	77	1.13	-2	0.99	14	40%	35	489	1865	1.15	-2	0.98	20	55%	36	717
Oroville_S	40	1.16	-5	1.00	34	31%	111	852	1016	1.19	-7	0.99	49	48%	103	1506
Pinehurst_S	6	0.95	5	0.61	5	35%	13	17	157	0.85	7	0.54	7	53%	13	43
RTP_S	17	0.81	2	0.88	2	25%	7	13	429	0.93	2	0.88	2	31%	7	40
Tulelake_S	78	1.02	3	0.98	6	27%	23	213	1720	0.98	4	0.95	10	45%	23	422
All smoke	729	0.95	3	0.97	20	41%	50	887	18082	0.99	0	0.95	27	55%	49	1506
ALL	4964	0.95	0	0.97	7	54%	14	887	126148	1	0	0.96	10	73%	14	1506

7 Additional analysis on missing RH

A brief analysis was done to understand how the performance would change for sites without RH. Overall, the agreement by AQI category is similar (within 3%) whether the RH measurements or an assumed default of 50% RH is used, however, using the RH does allow the correct AQI category to be more often predicted (94% of the time with RH, 93% of the time without).

Table S 15**.** Percent of PurpleAir NowCast AQI categories in agreement with monitor NowCast. Compares the final corrected PurpleAir data with the transition to assuming relative humidity levels of 0, 50, and 100%. Green shaded cells indicate better performance (a larger percentage of NowCast averages correctly reported) than the final correction while grey shaded cells perform worse than the final correction with the transition.

Reference AQI category	Category difference	Count in category (N)	Final correction (%)	Final correction assume 50% RH (%)	Final correction assume 0% RH (%)	Final correction assume 100% RH (%)
Good	0	136612	98	97	85	100
Good	1		2	3	15	0
Good	2		0	0	0	0
Moderate	0	21363	71	71	87	45
Moderate	-1		25	26	6	54
Moderate	1		4	3	7	1
Moderate	2		0	0	0	0
UHSG	0	1803	66	69	63	63
UHSG	-1		8	11	4	25
UHSG	1		25	21	33	12
UHSG	-2		0	0	0	0
Unhealthy	0	1982	94	93	93	89
Unhealthy	-1		3	4	3	8
Unhealthy	1		3	3	5	2
Unhealthy	-2		0	0	0	1
Very Unhealthy	0	615	89	88	89	87
Very Unhealthy	-1		7	7	5	10
Very Unhealthy	1		5	4	6	3
Hazardous	0	969	88	88	89	86
Hazardous	-1		12	12	11	14
All Categories	0	163344	94	93	85	92
All Categories	-1		4	4	1	8
All Categories	1		3	3	14	1
All Categories	-2		0	0	0	0
All Categories	2		0	0	0	0
